# A randomised phase II trial of Stereotactic Ablative Fractionated radiotherapy versus Radiosurgery for Oligometastatic Neoplasia to the lung (TROG 13.01 SAFRON II)

**DOI:** 10.1186/s12885-016-2227-z

**Published:** 2016-03-04

**Authors:** Shankar Siva, Tomas Kron, Mathias Bressel, Marion Haas, Tao Mai, Shalini Vinod, Giuseppe Sasso, Wenchang Wong, Hien Le, Thomas Eade, Nicholas Hardcastle, Brent Chesson, Daniel Pham, Morten Høyer, Rebecca Montgomery, David Ball

**Affiliations:** Peter MacCallum Cancer Centre, 2 St Andrews Place, East Melbourne, 3002 Australia; University of Melbourne, Royal Parade, Parkville, 8006 Australia; Centre for Health Economics Research and Evaluation, University of Technology Sydney, PO Box 123, Broadway Sydney, 2007 Australia; Princess Alexandra Hospital, Ipswich Rd, Woolloongabba, Qld 4102 Australia; Cancer Therapy Centre, Liverpool Hospital, Locked Bag 7103, Liverpool BC, NSW 1871 Australia; Radiation Oncology Department, Auckland City Hospital, Auckland, New Zealand; Department of Radiation Oncology, Prince of Wales Hospital, Barker St, Randwick, NSW 2031 Australia; Department of Radiation Oncology, Royal Adelaide Hospital, North Terrace, Adelaide, SA 5000 Australia; Department of Radiation Oncology, Northern Sydney Cancer Centre, Royal North Shore Hospital, St Leonards, 2065 NSW Australia; Department of Oncology, Aarhus University Hospital, Aarhus, 8000 Denmark; Trans Tasman Radiation Oncology Group (TROG), PO Box 88, Waratah, 2298 Australia

**Keywords:** SBRT, SABR, Metastases, Lung, Cost effectiveness, Quality of life

## Abstract

**Background:**

Stereotactic ablative body radiotherapy (SABR) is emerging as a non-invasive method for precision irradiation of lung tumours. However, the ideal dose/fractionation schedule is not yet known. The primary purpose of this study is to assess safety and efficacy profile of single and multi-fraction SABR in the context of pulmonary oligometastases.

**Methods/Design:**

The TROG 13.01/ALTG 13.001 clinical trial is a multicentre unblinded randomised phase II study. Eligible patients have up to three metastases to the lung from any non-haematological malignancy, each < 5 cm in size, non-central targets, and have all primary and extrathoracic disease controlled with local therapies. Patients are randomised 1:1 to a single fraction of 28Gy versus 48Gy in four fractions of SABR. The primary objective is to assess the safety of each treatment arm, with secondary objectives including assessment of quality of life, local efficacy, resource use and costs, overall and disease free survival and time to distant failure. Outcomes will be stratified by number of metastases and origin of the primary disease (colorectal versus non-colorectal primary). Planned substudies include an assessment of the impact of online e-Learning platforms for lung SABR and assessment of the effect of SABR fractionation on the immune responses. A total of 84 patients are required to complete the study.

**Discussion:**

Fractionation schedules have not yet been investigated in a randomised fashion in the setting of oligometastatic disease. Assuming the likelihood of similar clinical efficacy in both arms, the present study design allows for exploration of the hypothesis that cost implications of managing potentially increased toxicities from single fraction SABR will be outweighed by costs associated with delivering multiple-fraction SABR.

**Trials registration:**

ACTRN12613001157763, registered 17th October 2013

## Background

SABR is emerging as a non-invasive method for precision irradiation of pulmonary oligometastases using radioablative doses with a higher biological effect than can be achieved with conventional radiotherapy. The paradigm of aggressive local treatment with SABR for oligometastatic disease is well recognised [1–3]. Stereotactic body ‘radiosurgery’ (SRS) refers to the accurate delivery of a single precise, large and highly conformal SABR treatment. Multi-fraction SABR and single fraction SABR represent a radical departure from classical fractionated radiotherapy. A previous systematic review [4] of SABR for secondary lung cancers performed in 2010 revealed 154 patients treated with single fraction SABR and 343 patients treated with fractionated SABR. In the single fraction experience, the mean weighted 2 year local control was 78.6 % (range 48–91 %) and 2 year overall survival was 50.3 % (range, 33–73 %). The rate of significant toxicity (grade 3 or higher) was only 3.3 %. The results are comparable in the fractionated SABR series. The 2-year weighted local control was 77.9 % (range, 67–96 %). The corresponding 2-year weighted overall survival was 53.7 % (range 33–89 %), with a 4 % rate of grade 3 or higher radiation toxicities. These outcomes are comparable with surgical alternatives, with low rates of significant toxicity.

Stereotactic radiotherapy is a rapidly evolving technique that has been implemented widely through Europe, North America and Japan. A survey of 1600 American radiation oncologists showed that 64 % of physicians used SABR (95 % confidence interval, 60–68 %), of whom nearly half adopted it in 2008 or later [5]. Lung was the most popular site of SABR use (89 %), with the three and four fraction SABR schemes accounting for 68 % of prescribed treatments. In contradistinction the single fraction approach is commonly employed by several institutions in Europe [6–10]. Similarly in the Australian context several dose-fractionation schedules have been developed. For example the Peter MacCallum Cancer Centre in Victoria has reported the use of a single fraction technique [11], whereas the Northern Sydney Cancer Centre have implemented a four fraction SABR approach in New South Wales. A retrospective comparison of these two approaches indicated no significant differences in clinical outcomes between single or multi-fraction approaches [12].

The primary purpose of this study is to compare single versus multi-fraction SABR in the context of pulmonary oligometastases. The proposed investigational fractionation schedules in the SAFRON phase II study are 28Gy in one fraction versus 48Gy in four fractions of SABR. Both fractionation schedules have been previously used in the context of lung metastases [4]. Comparing these arms using the biological effective dose (BED) calculation [13], it is apparent that these fractionation schedules are very similar for tumour effects (Table 1). Both arms deliver biological effective doses above 100Gy to the periphery of the target, which is known to correlate with very high rates of local control in the order of ~90 % [6, 10, 14]. A single fraction SABR is theoretically as effective as four-fraction SABR and is more convenient for the patient and has the potential to be more cost-effective. However the BED calculations (Table 1) suggest that there is a potential for greater late tissue toxicity from this approach. Theoretically, much of this potential toxicity is mitigated by highly accurate radiation delivery; nevertheless, there is clear clinical and theoretical equipoise to support the design of this trial.

**Table 1:** BED calculations

	Arm (1): 28Gy in 1#	Arm (2): 48Gy in 4#
*Early (tumour) effects α/β = 10*	106Gy	105Gy
*Late (tissue) effects α/β = 3*	289Gy	240Gy

## Methods/Design

### Study design

**Fig. 1:**
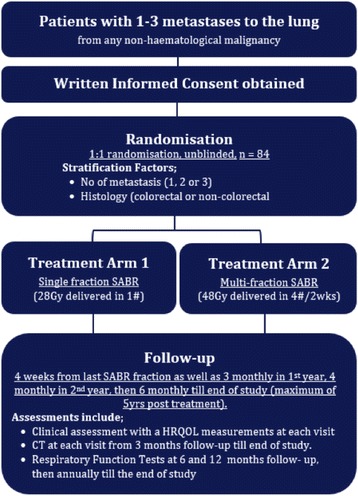
Study flowchart

This study is lead by the TransTasman Radiation Oncology Group (TROG) in collaboration with the Australasian Lung Cancer Trials Group (ALTG). The TROG 13.01/ALTG 13.001 SAFRON II study is a multi-institutional randomised interventional phase II clinical trial. The study has ethics board approval from the Peter MacCallum Cancer Centre (HREC/14/PMCC/2), and is registered on www.clinicaltrials.gov (ID: NCT01965223). All participating centres will obtain ethical approval prior to study activation. The study population are patients with oligometastases (1–3 metastases) to the lung (from any non-haematological malignancy). The trial schema can be found in Fig. 1. The intervention for ARM 1 is single fraction SABR - 28Gy delivered in one fraction. The intervention for ARM 2 is multi-fraction SABR - 48Gy delivered in four fractions, delivered over 2 weeks, with each fraction on non-consecutive days. Table 2 outlines dose constraints. Follow-up clinical visits including surveillance CT scanning will occur 3 monthly for year 1, 4 monthly for year 2, and thereafter 6 monthly until year 5 after treatment delivery. Written informed consent will be obtained from all individuals for participation in this study.

**Table 2:** Normal tissue dose-volume constraints and standardised contouring nomenclature

Organ	Standardised name	Parameter	Investigational treatment	
		Constraint	28Gy in 1#	48Gy in 4#/2wks
Normal Lungs		V5 < 1000 cc	66 % 7.4Gy	66 % 12.4Gy, (max 3.1Gy per fraction)
Heart	Heart	Maximum dose (0.03 cc) < 15 cc	22Gy	34 Gy, (max 8.5 Gy per fraction)
			16Gy	28 Gy, (max 7 Gy per fraction)
Oesophagus	Oesophagus	Maximum dose (0.03 cc)	15.4Gy	30Gy, (max 7.5Gy per fraction)
Spinal Cord	SpinalCord	Maximum dose (0.03 cc)	12Gy	20.8Gy, (max 5.2Gy per fraction)
Brachial plexus	BrachialPlexus	Maximum dose (0.03 cc)	15Gy	24Gy, (max 6Gy per fraction)
Skin (5 mm subcutis)	Skin	Maximum dose (0.03 cc) < 10 cc	26Gy	36 Gy, (max 9 Gy per fraction)
			23Gy	33.2 Gy, (max 8.3 Gy per fraction)
Chest wall^a^	ChestWall	<70 cc	^b^26Gy to full thickness	30Gy
Great Vessels	GreatVessel	Maximum Dose (0.03 cc)	30 Gy	49 Gy, (max 12.25 Gy per fraction
Liver	Liver	V20, V30	No constraint, but dose/volume parameters to be documented	No constraint, but dose/volume parameters to be documented

^a^Chest wall dose limit may be exceeded if rib structure lies close to or in contact with the PTV

^b^26Gy isodose line should not cross full thickness of the chest wall structure

Definitions: Vx describes the volume that receives xGy, e.g. V5 < 66 % represents that the volume of specified OAR receiving 5 Gy shall be less than 66 %

The primary endpoint is safety of SABR treatment as measured by the incidence of grade 3 and 4 toxicities measured using CTCAE V4.0 within 12 months of treatment completion. Key secondary endpoints include a) Quality of life using EQ-5D and MDASI-LC, b) Local efficacy (time to local failure), c) Resource use and costs associated with treatment, d) Other clinical outcomes (overall survival, time to distant failure and disease free survival).

### Inclusion/exclusion criteria

Patients may be included in the trial only if they meet all of the following key inclusion criteria at randomisation:

- Aged 18 years or older
- ECOG 0–1 inclusive
- A maximum of three metastases to the lung from any non-haematological malignancy
- Individual tumour diameter ≤ 5 cm. Targets are located away from central structures (defined as 2 cm beyond bifurcation of lobar bronchi and central airways)
  - o Note: Targets in proximity to chest wall and mediastinum that meet these inclusion criteria are eligible
- Primary and extrathoracic disease controlled with local therapy (e.g. surgery/definitive radiotherapy)

Key exclusion criteria are listed below:

- Previous high-dose thoracic radiotherapy in region of proposed SABR, as defined as a BED_10_ of 40Gy
- Cytotoxic chemotherapy within 3 weeks of commencement of or concurrently with treatment
  - o Hormonal manipulation agents are allowable concurrently with treatment (e.g. aromatase inhibitors, selective oestrogen receptor modulators, and gonadotrophin releasing hormone receptor modulators)
- Concurrent targeted agents (such as sunitinib, bevacizumab and tarceva) are not allowed
- It is recommended that targeted agents not be delivered within 7 days of delivery of radiation therapy treatment
- Germ cell and small cell carcinoma histologies

### Statistical considerations

This study is a randomised controlled phase II multicentre trial, with the main objective to determine whether single fraction radiosurgery (28Gy/1) or fractionated SABR (48Gy/4) has acceptable toxicity for the treatment of pulmonary oligometastases, as defined by a maximum acceptable toxicity rate of grade 3 or higher adverse events of < 5 %.)

If both treatments have acceptable toxicity profile the criteria for selecting which arm will be used in the phase III trial is as follows:

- The arm with superior clinical outcomes will be chosen, based on time to local failure and overall survival.
- If there is no significant difference in clinical outcomes, the arm with the superior quality of life will be chosen.
- If there is no significant difference in quality of life, the arm associated with the least amount of resource use will be chosen.

### Statistical analysis

Toxicities will be summarised as counts and percentages and presented in tabular form. The rate of grade 3 or higher toxicities at 1 year will be estimated for each arm by two methods. The first method (primary analysis) will evaluate the toxicities for participants who complete 1 year of follow up with 80 % confidence interval assuming binomial distribution. The second method (sensitivity analysis) will estimate the toxicities using cumulative incidences with death as a competing event. Time to event outcomes will be described using Kaplan-Meier methods with 95 % confidence intervals for each arm. The curves will be compared between arms using the Logrank test.

Overall Health-Related Quality of Life (HRQOL) will be measured using the MDASI-LC, a 22 item QoL module with three specific lung cancer questions. The results will be analysed using general linear mixed models. The area under the curve (AUC) will be compared between the arms using linear contrasts from the general linear mixed model. The linear mixed model will include arm, time and the interaction between arm and time as fixed effects with patients as random effect. Baseline MDASI-LC will be included as a covariate in the model. Completion rate of MDASI-LC at each time point will be reported in tabular form.

### Health economics: assessment of quality of life

For the purposes of the economic evaluation, HRQOL will be assessed using the EQ5D-5 L, a validated self-completed multi-attribute utility measure which will be used to estimate Quality adjusted life years (QALYs) for use the economic evaluation [15, 16]. This 5 item scale covers the following dimensions: mobility, self-care, usual activities, pain/discomfort and anxiety/depression, with each dimension having five levels. Participants will complete both measures at baseline and during follow-up.

### Health economics: assessment of resource use and costs

The direct resources associated with the delivery of SABR will be measured by observing the health care professional time and consumables required to plan and deliver of the radiotherapy intervention; an average cost/fraction will be calculated, reflecting Australian equipment and practice. Patient costs will be measured in terms of travel time and clinic time (i.e. time away from usual activities). Any visits to Emergency Departments or admissions to hospital will be captured directly from hospital records. Consent will be obtained from participants to access their Medicare Benefits Scheme (MBS) and Pharmaceutical Benefits Scheme (PBS) claims through Medicare (or appropriate national health administration) in order to capture any ancillary health expenses not costed directly to the primary cancer hospital provider. Hospital-specific costs and market prices are likely to be available for most of the resource items (e.g. MBS-fees). In the absence of market prices, data from the literature and expert opinion will be used to estimate unit prices. Results will be presented as total health care (by type) used, cost per unit of health care (e.g. PBS price) and total cost of health care used over the period of the trial and follow-up (3-years).

### Health economics analysis

The results of the economic evaluation will be reported as net costs and benefits for ARM 1 versus ARM 2. The costs of each ARM will take into account any cost-savings due to avoided health care utilisation and/or toxicities. Mean estimates of costs will be used and confidence intervals will be generated by boot-strapping the data. Benefits will be measured via the EQ5D5L questionnaire. Results will be presented in terms of the incremental cost-effectiveness ratio (ICER) as a cost/QALY gained. The incremental QALY will represent the improvement in quality of life between ARM 1 and ARM 2. The robustness and validity of the cost-effectiveness analysis will be explored using probabilistic sensitivity analysis.

### Sample size calculation

The maximum acceptable toxicity rate (grade 3 or higher adverse events) is considered to be 5 %. The desired upper limit of the 80 % confidence interval for a true toxicity rate of 5 % is 17 %. For a one-sided exact test for proportion with alpha = 0.1 and 80 % power, the required sample size is 38 evaluable patients for each arm of the trial. Assuming that up to 8 % of participants may be considered ineligible/unanalysable, 42 participants will be recruited in each arm.

### Facilitating multicentre implementation of SABR through an online e-Learning platform

Online platforms are useful tools for teaching, training and education of health care professionals. There are a limited number of studies examining web-based training for radiation oncology clinicians [17, 18]. There has also been description of how to design a radiation oncology curriculum for e-Learning [19]. Prior to this study, there has been only one previous report of the use of e-Learning to support advanced image guided multi-centre radiotherapy trials [20]. As part of the TROG 13.01/ALTG 13.001 study, radiation therapists (RT), radiation oncologists (RO) and radiation oncology medical physicists (ROMP) are required to complete an e-Learning package to facilitate the safe and effective delivery of SABR to the lung. The e-Learning material covered 7 modules: Clinical Background, Organs-at-risk Contouring, Planning Technique & Evaluation, plan optimisation, Patient Specific Quality Assurance, 4DCT Simulation and CBCT & Image Guidance. The modules were created by a multi-disciplinary team consisting of radiation oncologists (RO), radiation therapists (RT), diagnostic radiologists and medical physicists (ROMP). As part of the credentialing processit was a requirement for RT, RO and ROMP to complete a subset ofcore modules that were specific to their profession (listed in Table 3). There were no requirements to complete non-core modules however these were available for participants to complete. The objective of this study platoform was to improve confidence and increase operator knowledge in lung SABR through the use of pre- and post-test assessment as well as long-term retention assessment.

**Table 3:** e-Learning module description and discipline-specific ‘core modules’ allocated for completion

Modules	RO	RT	ROMP
Clinical Background	✓	✓	✓
Contouring Organs-at Risk *(not included in the tests)*			
Planning Technique & Evaluation	✓	✓	
Planning Optimisation	✓	✓	
Patient Specific QA *(not included in the tests)*			✓
4DCT Simulation		✓	✓
CBCT & Image Guidance	✓	✓	✓

### Translational substudies

Out-of-field tumour regression (the Abscopal effect) is a known systemic effect of radiation in the preclinical and clinical setting [21, 22]. Direct ionising radiation elicits innate immune recognition of tumour, in the absence of a pathogen, through the liberation of cellular stress signals collectively termed, “danger signals” [23, 24]. The primary driver of increased immune mediated cell death is an enhanced capacity to recognise and mount an adaptive immune response to the established tumour. Three molecular signals are primarily responsible: the promotion of uptake of dying cells by dendritic cells (DCs), the cross-presentation of tumour-derived antigens to T cells and the activation of anti-tumour T cells [25]. These responses provide tools for improved recognition and killing by tumour-antigen reactive T cells [26]. There is presently no clinical data in humans assessing the effects of fractionation in ablative radiotherapy. As part of this study, peripheral blood mononuclear cells will be collected in a subset of patients prior to and after delivery of SABR. Where possible, pre and post-treatment tumour biopsy will be performed in this subset. A comprehensive characterisation of lymphocyte populations residing within the tumour bed and peripheral blood is planned. In addition to the planned substudy assessing immune effects of SABR, trial datasets will be available for secondary analysis of post-hoc technical and clinical objectives through TROG.

## Discussion

SABR is a non-invasive alternative treatment option that is presently available for a variety of pulmonary malignancies. In the setting of primary NSCLC, it has been postulated that SABR may be more desirable in patients with pre-existing comorbidities and the elderly [27]. Indeed, controlled non-randomised series also suggest SABR may be a valid approach in patients considered operable with similar outcomes to surgery in Stage I NSCLC [28]. More recently, the combined results of the ROSEL and STARS randomised clinical studies demonstrates clinical outcomes from SABR that compare favourably with surgery with fewer associated toxicities in operable patients with stage I NSCLC [29]. However, it is unclear at present what the ideal approach for dose/fractionation should be in the setting of primary NSCLC. The RTOG 0915 study demonstrated comparable clinical outcomes of a single fraction of 34 Gy approach in comparison to 48Gy delivered over four fractions [30]. There was a pre-stated plan for comparison with the standard of 54 Gy in 3 fractions used in the United States for stage I NSCLC, however this phase III study may not eventuate.

The management of patients with distant metastases from solid tumours is usually conducted with palliative intent. On analysis of the Surveillance, Epidemiology, and End Results (SEER) database, the 5-year survival of patients with metastatic disease of common malignancies such as colorectal, breast and lung cancer was 7, 19 and 2 % respectively [31]. Treatment predominantly involves palliative chemotherapy to address widespread disease without expectation of long-term survival. In contrast, Hellman and Weichselbaum hypothesised the existence of an intermediate state between widespread metastatic disease and locally confined disease and coined the term “oligometastasis” [1]. In this setting, targeted therapies have procured significant long-term survival. Surgical resection has been shown in a randomised trial to increase median survival in patients with single brain metastases from 15 weeks to 40 weeks (*p* = 0.01) [32]. Systematic reviews of the resection of hepatic metastases show a 5-year survival of 25–30 % [33, 34]. Pulmonary tissue represents a common site for metastatic seeding. A multinational registry of 5206 patients undergoing surgical resection of lung metastases showed a 5-year survival rate of 36 %, with the median survival being 35 months [35]. All these results are remarkable given the typically poor survival for patients with metastatic tumours, and may justify an aggressive approach for patients with ‘oligometastatic’ disease. However, metastasectomy can be associated with significant risk of patient morbidity and the cost-effectiveness of such an approach is currently unknown.

Very little is known regarding the costs, quality of life or QALY outcomes secondary to SABR in the setting of pulmonary metastatic disease. Shared decision-making is now advocated as the preferred model of treatment planning [36]. In order to make informed treatment decisions, patients and clinicians need to know possible adverse effects on quality of life; particularly if the patient has considerable co-morbidities as is commonly the case in an ageing population. Given the explosion of trials internationally investigating SABR, it is surprising that there has been little effort to formally assess quality of life outcomes of SABR. As the primary objective is to determine whether SABR can be delivered safely with minimal toxicity, the participants in this trial randomised to a single fraction treatment may perceive that the benefit of faster, convenient treatment delivery comes at the cost of an increased likelihood of sustaining toxicity. However, expected toxicities are low in comparison to surgical lung resection, where rib spreading procedures result in up to 44 % of patients suffering pain longer than 6 months after surgery [37] and approximately 30 % of patients suffering chronic pain beyond 5 years after surgery [38]. Hence, less arduous SABR treatment techniques with expected high levels of cancer control are likely to produce low levels of psychological distress, particularly anxiety. This information may be used to inform the design of future randomised phase III studies.

The full immunological potential of radiotherapy may be influenced by the dose and fractionation of radiation employed, for both single fraction and fractionated approaches [39]. Ablative dose ranges employed by SABR heralds a potential for even greater augmentation of the tumouricidal immune response than conventional radiotherapy [40]. Ablative doses result in a greater degree of stromal-vascular damage, ceramide-induced endothelial cell damage which may result in complete inhibition of tumour re-vascularisation and increased apoptosis of tumour cells [41–43]. Immunogenic responses at sites distant to the SABR therapy have already been reported by our group [44] and others [45]. It is unclear, however, whether single fraction or hypofractionated radiotherapy is optimal in eliciting immune responses. For example, significant cross-priming of T-cells against tumour antigens have been demonstrated to be induced by a single dose of 15Gy in the draining lymph nodes [46]. Our group at the Peter MacCallum Cancer Centre identified that single dose (12Gy) radiotherapy did not deplete established tumours of effector cells critical to the anti-tumour activity with enrichment of functionally active, tumour-specific T-cells [47]. This is similar to a recent demonstrating that single fraction of 30Gy to tumour resulted in an intense activated T cell tumour infiltrate, and a loss of myeloid derived suppressor cells [48]. Single fraction ablative RT has also been shown to synergise with the T-cell checkpoint inhibitor anti-PD-1 in murine models allowing for induction of an anti-tumour immune response by relief of tumour-mediated immunosuppression [49–51]. On the other hand, reports from New York University suggest that 3 × 8Gy hypofractionated radiotherapy results in enhanced immunogenicity in direct comparison to single fraction ablation [52, 53]. The translational component of this study aims to help better define the innate immune response evoked by different fractionation schedules of SABR and to investigate the prognostic implications of these changes. In the future this research may help to define strategies for combining immunotherapy to maximise patient outcomes after SABR.

Fractionation schedules have not been investigated in a randomised fashion to date in the setting of oligometastatic disease. From a philosophical perspective, use of the fewest possible treatments whilst maintaining clinical effectiveness is critical in this cohort of patients given the significant competing risks of distant disease recurrence. The present design allows for exploration of the hypothesis that cost implications of managing increased toxicities from single fraction SABR may be outweighed by costs associated with multiple-fraction SABR, with the assumption of similar clinical efficacy in both arms.

## Abbreviations

4D: four-dimensional
ALTG: Australasian Lung Cancer Trials Group
BED: biological effective dose
CBCT: cone beam CT
CT: computerised tomography
CTCAE: common terminology criteria for adverse events
ECOG: Eastern Co-operative Oncology Group
HREC: Human Research Ethics Committee
HRQOL: health-related quality of life
ICER: incremental cost effectiveness ratio
MBS: medical benefits system
MDASI-LC: M.D. Anderson Symptom Inventory – Lung Cancer
PBS: pharmaceutical benefits scheme
QA: quality assurance
QALY: quality-adjusted life year
SABR: stereotactic ablative body radiotherapy
SRS: stereotactic body radiosurgery
TROG: Trans Tasman Radiation Oncology Group
